# Determining optimal neighborhood size for ecological studies using leave-one-out cross validation

**DOI:** 10.1186/1476-072X-11-10

**Published:** 2012-04-03

**Authors:** Deok Ryun Kim, Mohammad Ali, Dipika Sur, Ahmed Khatib, Thomas F Wierzba

**Affiliations:** 1 International Vaccine Institute, San 4-8, Nakseongdae-dong, Gwanak-gu, Seoul, South Korea; 2 National Institute of Cholera and Enteric Diseases, Beleghata, Kolkata 700 010, India; 3 Ministry of Health and Social Welfare, P.O.Box 236, Zanzibar, Tanzania

**Keywords:** Neighborhood, Ecological study, Leave-one-out cross validation

## Abstract

We employed a leave-one-out cross validation to determine optimally sized neighborhood. Variations between a single point and the other points within each filter size for all the points in the study area were evaluated, and the mean squared error (MSE) for each filter was calculated. The filter with the lowest MSE was considered as the optimal neighborhood. The method is useful in determining the optimal neighborhood for both geographic and population filters.

## Background

There has been a growing interest in ecological research assuming that the neighborhood where people reside may influence their health status [1,2] as much as personal risk factors like age and gender [3-6]. Several factors have stimulated interest in ecological research. Chief among them are studies of the determinants of health [7,8] which recognize that social influences on health operate through neighborhoods [1,2]. The emergence of multilevel analytical methods permits investigators to quantify the health effects of neighborhoods [8,9]. The growing concern about confidentiality of individual health data [10,11] has also motivated investigators to examine alternative methods for observational studies. Interest in ecological research is also due to ease, as risk factors are often not available at the individual level or may not be obtained or acquired from pre-existing sources (e.g., census or aggregated survey data).

An ecological variable most commonly refers to a set of individual level attributes which have been aggregated up to the areal level (e.g., percent poor, diabetes prevalence). Defining the neighborhood boundaries in which variables are aggregated greatly influences the results of statistical analyses [12] due to the modifiable areal unit problem (MAUP). The MAUP is a potential source of error, in particular when point-based measures of spatial phenomena are aggregated to an aerial unit which can affect spatial studies that utilize aggregate data sources [13]. For example, census data may be aggregated into census enumeration districts, or villages, communities, or any other spatial partition, thus, the areal units are modifiable. The MAUP problem has been addressed in the area of spatial crime analysis, where the areal units used in many geographical studies are arbitrary, modifiable, and subject to the whims and fancies of whoever is doing, or did, the aggregating [14]. However, the implications of aggregating strategies are often overlooked [15,16]. For example, administrative units have been used as proxies for neighborhood when aggregating socioeconomic variables. Despite the convenience of the administrative units for statistical analyses, several researchers have questioned whether an administrative unit is sufficiently consequential to impact health status [16-18].

Boyle and Willm (1999) suggested that the salient features of ecological variables must be defined so that spatial aggregations can be configured and sample populations identified that adequately represent influences on human behavior [16]. The question of what constitutes a meaningful neighborhood is largely dependent on the research question and is particularly important when one seeks to examine neighborhood effects on health. For example, neighborhoods defined on the basis of people's perceptions may be relevant when the neighborhood characteristics of interest relate to social interaction or social cohesion, administratively defined neighborhoods may be relevant for policies, and geographically defined neighborhoods may be relevant for features of the chemical or physical environment [19].

Administratively-based neighborhoods may also be too large to evaluate the effect of context on health or health-related behavior. This kind of neighborhood may be arbitrary with respect to health [20] and may not capture contextual information for those individuals residing on the edge of adjacent neighborhoods [21]. Also, the ecological processes do not necessarily recognize the artificial administrative-based boundaries. Neighborhood also has a social as well as spatial dimension, and a geographically smaller neighborhood often provides a more accurate measurement of neighborhood effects [12]. In a small neighborhood, the outcome of aggregation may be unstable. The size of neighborhood may vary according to the processes through which the neighborhood effect is hypothesized to operate and the outcome being studied [22]. The correct level of aggregation of socioeconomic ecological variables using the neighborhood approach is the subject of ongoing research [8].

There are several methods that can be used to determine the optimal neighborhood size for risk-factor aggregation. Kelsall and Diggle [23] used kernel smoothing to select an optimal scale using weighted least squares cross-validation that minimizes an average squared predictive error criterion at every point *i *in the data set using the fitted value obtained by leaving the point *i *out of the sample. Minimizing this criterion over many possible scales is very computationally intensive because the model is fitted *n *times for each scale choice, where *n *is the number of points in the data set. Wood [24] uses a less computationally intensive function to estimate smoothing parameters in generalized ridge regression with multiple penalties using generalized cross validation. Webster et al. [25] chose optimal scale by minimizing the Akaike's Information Criterion or AIC [26]. In this paper, we applied the leave-one-out cross validation with mean squared difference statistics to determine optimal neighborhood for aggregating individual level data on vaccine uptake. The leave-one-out is a special case of the general class of cross-validation error estimation method. It is an unbiased estimator of the true error rate of a classifier [27]. Evidence of the superiority of the leave-one-out is well documented [28]. The leave-one-out cross-validation involves using a single observation from the original sample as the validation (test) set, and the remaining observations as the training set. This is repeated such that each observation in the sample is used once as the validation data. This is the same as a *K*-fold cross-validation with *K *being equal to the number of observations in the original sample.

## Methods

### The study area and data

We used vaccine uptake data from a cholera vaccination program in Zanzibar, an archipelago approximately 50 km off the eastern coast of mainland Tanzania, consisting of two main islands, Unguja and Pemba [29] and from a phase IV typhoid vaccine effectiveness trial conducted in an urban slum of Eastern Kolkata [30]. Demographic, socioeconomic, and geographic information of all residents in the study area of the three sites were collected by project staff. Households of eligible individuals were referenced geographically and households sharing a single structure or closely connected structures were referenced as a single geographic point of residence (dwelling unit). There were 32,254 eligible individuals referenced by 4,479 geographic points of residence in Unguja, 15,925 eligible individuals referenced by 2,903 points of residence in Pemba, and 62,756 eligible individuals referenced by 10,415 geographic points of residence in Kolkata. Vaccine coverage was 44%, 61%, and 60% in Unguja, Pemba, and Kolkata, respectively (Table 1).

**Table 1:** Characteristics of the three study sites

Site	Area (km^2^)	Population density (100 m^2^)	CV of population density from 10-200 m radius, incremented by 10. Mean (Std.)	# geographic points of residence	# eligible population	# Vaccine recipients	Vaccine coverage (min. - max.)
Unguja	3.17	101.5	0.36 (0.10)	4,479	32,254	14,107	44 (0-100)

Pemba	27.58	5.77	0.59 (0.03)	2,903	15,925	9,732	61 (0-100)

Kolkata	0.99	633.90	0.73 (0.06)	10,415	62,756	37,673	60 (0-100)

*CV *coefficient of variation; *Std*. standard deviation; *Min*. minimum vaccine coverage by geographic points of residence; *Max*. maximum vaccine coverage by geographic points of residence

### Filtering methods

There are three filtering methods by which one can define the optimal neighborhood. The three methods are: i) fixed geographic area, ii) nearly fixed population, and iii) fixed population. A fixed geographic area filtering method captures all individuals within a fixed area. Nearly fixed population accumulates individuals from the nearest geographic points to a predefined center point of the neighborhood until the individuals are greater than or equal to the target size of the population in the neighborhood. In this case, the number of individuals in the neighborhood may exceed the target size of population if the last geographic point of residence adds more individuals than are needed to reach the target size of population. Fixed population is very similar to nearly fixed population with the exception that if the number of individuals exceeds the target population when the last geographic point of residence is captured, then a proportion of the individuals of that point are included to maintain exact target population. Out of the three filtering methods, we tested fixed geographic filter and nearly fixed population filter in our exercises. We did not test the fixed population filter in our exercises because it produces similar results (from our experience) to the nearly fixed population. Besides, we believe nearly fixed population is more practical than fixed population as segregating the proportion of the people of the last geographic point is arbitrary.

### Leave-one-out cross validation

In this leave-one-out cross validation, we defined the test set using the individuals living in a geographic point of residence and the training set using the individuals from the remaining points of residence within the filter. This allowed us to select the optimal neighborhood by minimizing the mean squared difference of the data obtained from the test set and the training set under the assumption that the data are more alike within the filter than across the filters. For each geographic point of residence, we aggregated vaccine uptake within the selected size of the filter.

Since contextual data using geographic filters may be affected by the heterogeneity of population distributions [31], we tested the leave-one-out cross validation approach on various spatial distributions of population. The variations of spatial distribution of population in the three sites were measured by the coefficient of variation (CV) of population density. As the first step, we computed Euclidean distances of all pairs of geographic points of residences in the study area. We arbitrarily set the minimum size to 50 m for the geographic filter and to 50 individuals for the population filter, and the maximum size to 2,000 m for the geographic filter and to 6,000 individuals for the population filter. We also arbitrarily set increments of 50 m when expanding geographic filter size from 50 m to 200 m and of 200 m when expanding from 400 m to 2,000 m. We used increments of 50 for population filter sizes between 50 and 200 and increments of 200 when expanding population filter size from 400 to 6,000 individuals. Since the population density of the Kolkata study site is much greater than the Unguja and Pemba study sites, we used increments of 10 m for geographic filter sizes varying between 10 m and 200 m. We determined the optimal neighborhoods for various spatial distributions of population of the study areas using both geographic and population filters.

### Steps of determining optimal neighborhood

The steps that we followed for determining the optimal neighborhood are as follows:

Step 0: *Initialization*

We set a number of filters *N_i _*(*i *= 1,2,..., k) with arbitrary sizes both for geographic and population filters. The size of radius is for the geographic filter and the size of population is for the population filter. The geographic points of residence were defined as *P_j _*(*j *= 1,2,..., n).

Step 1: *1^st ^Neighborhood (N_1_)*

**Figure 1:**
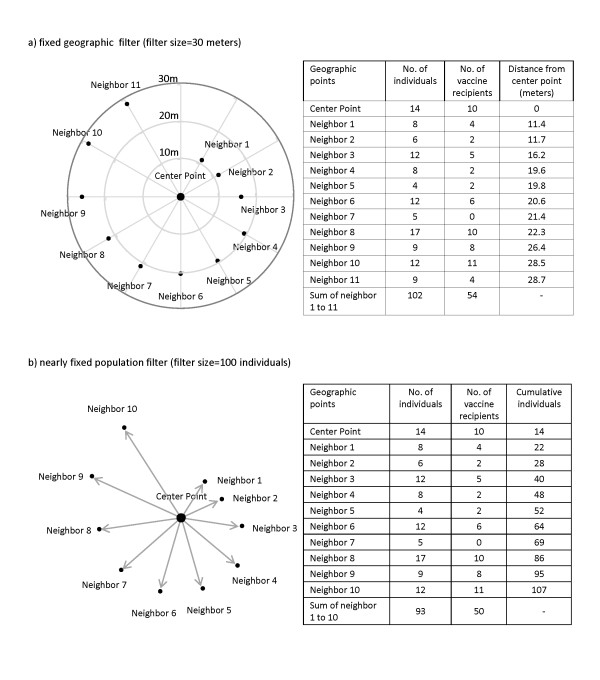
**An example of the fixed geographic and the nearly fixed population filters**.

We considered first the center geographic point of residence of the filter *N_1 _*and accumulated the individuals of the point for the test set. We then captured the 1^st ^nearest point of residence of the center point and accumulated the individuals living in that point for the training set of *N_1_*. We then moved to the 2^nd ^nearest point of residence of the center point and accumulated the individuals of the geographic point for the training set of *N_1_*, and so on. This process continued until all points within *N_1 _*were captured. An example of the filtering process by geographic and population filters is shown in Figure 1.

Step 2: *Estimation of Neighborhood N_1_*

The individuals living at the center point of residence of *N_1 _*were in the test set and the remaining individuals within *N*_*1 *_were in the training set as mentioned above. We then estimated vaccine coverage for the test set (*p_1_*) and the training set (*q_1_*).

Step 3: *Squared Error Calculation*

Based on the estimates *p_1 _*and *q_1 _*in step 2, we calculated squared error between *p_1 _*and *q_1_*.

Step 4: *New test and training sets*

We then moved to the 2^nd ^point of residence and repeated steps 1 through 3 for the same size of filter, *N_1_*. When all the test sets were evaluated for *N_1_*, we computed the mean squared error (MSE) using the following equation:

$$R_{loo\left(k\right)}^{\prime }=\frac{1}{n}\overset{n}{\underset{i=1}{\sum }}{\left(p_{i}-q_{i}\right)}^{2}$$

where,

*p_i _*= vaccine coverage from the test set of the filter *N_1_*,

*q_i _*= vaccine coverage from the training set of the filter *N_1_*,

n = total number of geographic points of residence in the study area.

Step 5: *Next filters (N_2_,..., N_k_) and the choice of optimal neighborhood*

We repeated steps 1 to 4 for the next filters, *N_2_,..., N_k_*. The mean of within-filter squared errors is the MSE of the leave-one-out cross validation. The process was repeated for various sizes of filter until the mean squared error was minimized. Finally, the filter that yielded the lowest MSE was chosen as the optimal neighborhood.

## Results

**Figure 2:**
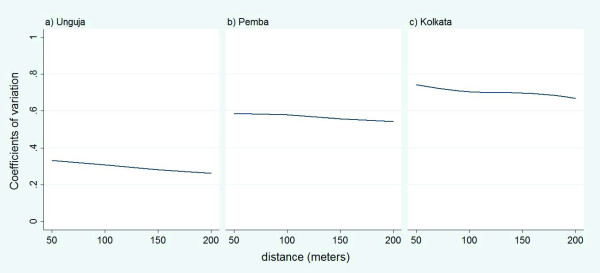
**Coefficient of variation (CV) of the population densities in the three study sites**.

The mean CVs of population density for different filters between 10 m and 200 m radius, with increments of 10 m, were 0.36, 0.59, and 0.73 for Unguja, Pemba, and Kolkata, respectively (Figure 2). Based on these CVs, we defined a homogeneous spatial distribution of population in Unguja, a moderately homogeneous spatial distribution of population in Pemba, and a heterogeneous spatial distribution of population in Kolkata

**Figure 3:**
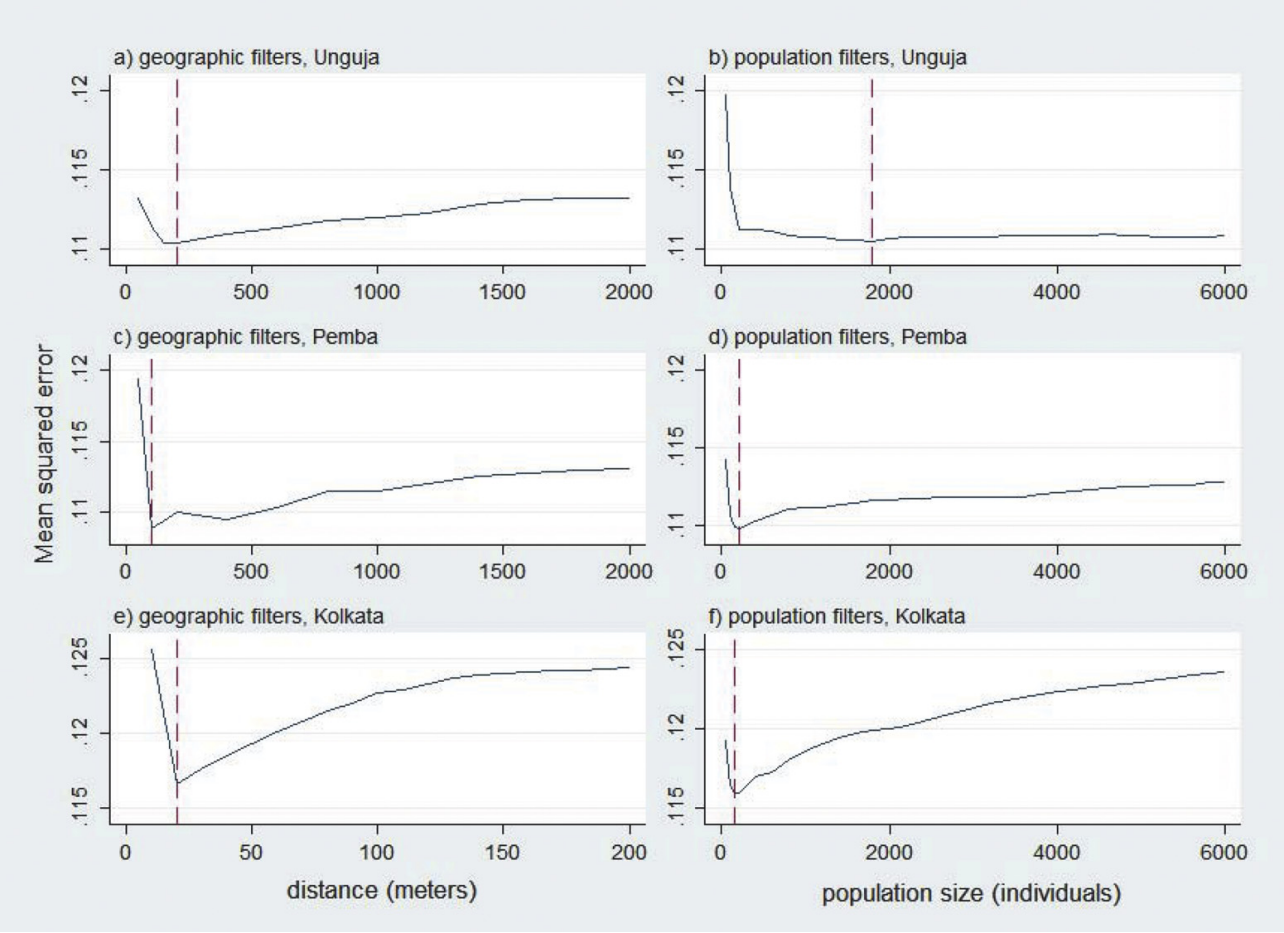
**Mean squared errors (MSEs) for geographic and population filters in the three study sites**.

The 200 m geographic filter had the lowest mean squared error (MSE) of vaccine coverage for Unguja (Figure 3). The average number of individuals in this optimally-sized neighborhood was 1,921 (Range: 50-3,151) (Table 2.). The population filter had the lowest MSE at 1,800 individuals; the average distance to achieve this size of population was 200 m. In Pemba, the optimal neighborhood had a 100 m radius with an average number of 158 individuals (range: 4-434). The optimal population filter included 200 individuals; the average distance to achieve this population was 158 m. In Kolkata, the optimal neighborhood is 20 m with an average of 283 individuals (range: 2-1,166) for the geographic filter. The optimal population filter included 150 individuals, requiring an average radial distance of 19 m.

**Table 2:** Optimal neighborhood size by geographic and population filters in the three study sites

Filter type		Unguja			Pemba			Kolkata	
	
	Size	Minimum mean squared errors	**Avg**. (min-max)	Size	Minimum mean squared errors	**Avg**. (min-max)	Size	Minimum mean squared errors	Avg. (min-max)
Geographic filter	200 m	0.1103	1,921* (50-3,151)	100 m	0.1089	158* (4-434)	20 m	0.1166	283* (2-1,166)

Population filter	1,800 people	0.1104	200 m** (141 m-1,005 m)	200 people	0.1098	158 m** (55 m-846 m)	150 people	0.1158	19 m** (0 m- 292 m)

*population, ** distance in radius

The optimal neighborhood obtained in Unguja and Kolkata for both geographic and population filters yielded same size of area (200 m for Unguja and ~20 m for Kolkata). In Pemba, the population filter yielded larger size of area than geographic filter in achieving optimal neighborhood (158 m vs. 100 m). The MSEs for both geographic and population filters are 0.110 in Unguja. In Pemba, the MSE for the geographic filter is 0.109, which is a bit lower than that for the population filter (0.110). And, the MSE for the geographic filter in Kolkata (0.117) is a bit higher than that for the population filter (0.116).

## Discussion

In our leave-one-out cross validation, we evaluated the variations in the data between a single point and the other points within each size of filter. This is done for all the points in the study area, and then we calculated a MSE for each filter size. The MSE defines the amount of variation for that specific filter size. Our results suggest that one can determine optimal neighborhood for ecological studies in health using the leave-one-out cross validation that measures contextual information within a circular area centered on the residences of individuals. This approach, which considers surrounding population rather than surrounding space, may be particularly appropriate when considering contextual factors in aggregating individual characteristics (e.g., vaccinated or not) rather than features of the physical environment [21]. The results of our method also suggest geographic or population filter can be used to determine the optimal neighborhood in a homogeneously spatial population distribution, but the population filter may provide better estimate if the spatial distribution of population is heterogeneous [31] like our Kolkata site. One potential problem of this approach is that it is very expensive from a computational point of view because of the large number of times the training process is repeated.

The results of our study indicate that understanding spatial variability of population is important for selecting a suitable filtering method for determining the optimal neighborhood. The limitation of our study is that we used an arbitrary cut-off for defining spatial variability of the population density in the study areas. Corbett and Jensen (1992) suggested a cut-off of CV < 0.5 for homogeneous density, CV > 0.5 and CV < 1.0 for heterogeneous density, and CV > 1.0 for very heterogeneous density [32]. In our case, we defined CV < 0.5 as homogeneous, CV > 0.5 and CV < 0.7 as moderately homogeneous, and CV > 0.7 as the heterogeneous, and believe that these definitions suited our study areas and are close to what has been reported in literature.

A practical use of this approach is to measure neighborhood level vaccine coverage for evaluating herd effect of a vaccine in an individually randomized trial, which has already been done in a study [33]. To measure the herd effect of a vaccine, it is necessary to evaluate the vaccine coverage among the individuals. If a person lives in a higher coverage neighborhood, certainly the risk of having the target disease will be lower for that person compared with the risk of a person living in a low coverage neighborhood. For this, an appropriate neighborhood of a household needs to be defined so that the contacts of the households are limited mostly within the neighborhood. Here, the neighborhood contacts are considered distance-based because the distance-based social connectivity was found to be more important than kinship-based social connectivity when evaluating transmission of an infectious disease [34]. If the neighborhood is small then we will miss potential contacts of the household, and if the neighborhood is large then we will add several non-contacts within the neighborhood. In both the cases, there will be dilution of the effect of intervention. Therefore, an appropriate size of neighborhood is important so that the potential contacts of a household can be captured within the neighborhood. The leave-one-out cross validation determines suitable neighborhood by looking at homogeneity of the characteristics of the people within the neighborhood. If people's characteristics are more homogeneous within the neighborhood, it may suggest strong social contact among people living in the neighborhood, and that is of importance for evaluating the impact of context of such neighborhoods on health outcomes.

## Conclusion

Use of the leave-one-out cross validation method to determine optimal neighborhood geographic or population size may benefit ecological studies in health. We discussed the potential utility of this method to calculate vaccine coverage in communities that have received vaccinations, as a first step to understanding herd immunity. Certainly this method can be used to create ecological risk factors for epidemiological studies. However, one should bear in mind that examining the role of optimal neighborhood characteristics is complex because many of these dimensions may be interrelated [35] and may also influence each other [1]. For example, features of physical environments of a neighborhood may influence the types of social interaction, and vice-versa

## Competing interests

The authors declare that they have no competing interests.

## Authors' contributions

DRK, MA and TFW contributed to the design and analysis of the study and writing of the paper. DS and AK contributed to the implementation and supervision of the study programs. All authors read and approved the final manuscript.
